# Proceedings of Atlanta Medical College

**Published:** 1867-12

**Authors:** 


### ARTICLE II.

### PROCEEDINGS OF ATLANTA MEDICAL SOCIETY.

February 12, 1867.

On the call for report of cases—

Dr. Armstrong reported a case of Puerperal Fever in a lady who had been living in a malarial region, and, after having suffered from intermittent fever, was left in an anæmic and feeble situation. In this condition, some two months ago, she was confined in childbed. Nothing unusual occurred during her confinement; but, some three weeks since, she exhibited symptoms of mania. For several days previously to her decided mental alienation, various household duties were occasionally improperly performed. Instead of pouring out a cup of coffee, she would sometimes fill the cup with water, without seeming to notice the mistake until informed of it. Would occasionally fall, as though suddenly paralyzed, when walking about.

While in this condition, and as well as usual for several days when going to bed, she awoke her husband at 4 o'clock in the morning, in a fit of wild delirium, which lasted during the day. Her bowels being constipated, fifteen grains each of calomel and jalap were ordered in the morning. During the afternoon, the bowels were thoroughly evacuated, and an opiate was ordered to be taken at night, with directions to give bromide potassa if she did not sleep. The following morning, I found her much better—almost entirely quiet; had slept under the influence of the opiate without any other anodyne.

A wet nurse was obtained for the child, and she put upon the use of tincture iron, wine and generous diet.

Dr. Johnson was of the opinion that puerperal mania might occur at any period of pregnancy, and at any time while nursing.

Dr. Stacy thought the case reported, in all probability, was the result of impoverished blood.

Dr. Goodman reported to the Society a case of monstrosity, of which a German woman, under his care, was delivered sometime since. The flexor muscles of one arm were permanently contracted; two fingers on each hand were webbed, and the anus occluded, the fæces passing through the vagina. A fluctuating tumor of considerable size existed on the head. The urethra was normal. It had very little inclination to nurse, and died at twelve days old, seemingly from inanition.

Dr. W. F. Westmoreland, on being asked his opinion as to the propriety of a surgical operation in this case, stated that, inasmuch as the bowels were freely evacuated through the vagina, there could be no absolute necessity for an immediate operation, and would not have advised it at that time.

Dr. O'Keefe had been impressed with the idea that more deformities were found in children of foreigners than native American women, and desired the opinion of members of the Society on this subject.

Dr. W. F. Westmoreland had frequently found foreigners the subjects of this misfortune, but thought, when compared with the same class of natives, it would not be found to occur more frequently amongst Germans, or those of any other nation coming to America.

Dr. W. F. Westmoreland reported a case in which serious symptoms resulted from slight injury to the ball of the middle finger of the left hand, by a brass pin. On the 30th of December last, the finger was slightly pricked near the end by a brass shawl-pin. No inconvenience attended it for two days, when a small collection of pus was found in the wound, and was evacuated two days in succession by lightly puncturing the cuticle. About the fifth day from receipt of the injury, darting pain in the wound, elbow, axilla, and in the region of the left paroted gland, was of frequent occurrence during the afternoon, with some general fever. The finger was swollen, tender and hot. These symptoms continued, and being thought indicative of approaching tetanus, at 9 o'clock in the evening, free incisions—transverse and longitudinal—were made in the finger, relieving promptly the nervous pain referred to. The inflammation continued, however, with fever of an intermittent character; the paroxysms ushered in, irregularly, with chills—sometimes violent rigors. Cool applications for awhile, succeeded by emolients to the finger, in a few days, moderated the local symptoms. The fever, however, continued despite every means adopted. Under the use of quinine, though repeatedly given in full doses, the fever became more continued, with dry tongue and slight delirium. Mercury and arsenic have been used without any perceptible relief.

The question arises, to what is the fever attributable? Is it the result of nervous derangement, produced by the local injury? The incisions made with the bistoury do not look healthy. The edges are everted, and fungous granulations shoot from their surfaces. Fever is now constant; pulse generally about eighty to the minute; skin most of the time dry; tongue brown and dry; very restless occasionally; no disposition to talk or take food; despondent; bowels somewhat constipated; complains of pain in the lower extremities.

Under all the circumstances, is it unreasonable to suppose that the shock given to the cerebro-spinal centres, though tetanus was prevented, has laid the foundation for this uncontrollable and protracted fever?

Dr. Johnson considered the case one of neglected intermittent fever, and was of the opinion that, with the use of stimulants, nourishing diet and the occasional administration of quinine and strychnine, a favorable result may be expected.

Dr. Stacy favored nourishing diet, and recommended, in addition to the other suggestions of Dr. Johnson, the use of iron by hydrogen as a hæmatic.

Dr. O'Keefe was unable to arrive at any very satisfactory conclusion as to the exact nature of the case under consideration; but, from the report made of it, and from his own knowledge of the symptoms, is inclined to the opinion that they are the result of injury received in the finger. Whether they are due to pyæmia or a direct impression made upon the nervous system, he was not at all satisfied, but thinks it not at all improbable that pyæmia, in a mild form, exists. He is inclined to think the treatment adopted in this case has resulted in aggravation of the symptoms, and that the course to be relied on, is that which is calculated to invigorate the declining energies of the system. Nourishing diet, and other supporting means, are certainly indicated.

Dr. Word was in doubt as to the character of the affection and its cause. He thought, however, from the symptoms detailed, and the general history of the case, that, though the existence of genuine typhoid fever may reasonably be inferred, it is not at all improbable that the low continued fever, and depressed condition of the vital energies, may be the result of ordinary intermittent fever. Symptoms of this kind he had recently met with in a case commencing with ordinary chills, in which turpentine and nourishing diet proved successful.

---

February 26, 1867.

On call for report of cases—

Dr. O'Keefe reported a case of labor with arm presentation. Was called to a woman thirty years old, in her fourth confinement, on Saturday at 3 P. M. The membranes had ruptured in the morning, when the pains ceased almost entirely; pulse 100. On examination, the arm of the child was found in the vagina. No movement had been felt by the mother for twelve hours, and the child was supposed to be dead. Delivery by turning was at once determined upon. An attempt to return the arm before introducing the hand into the uterus, was unsuccessful, when the left hand was carried into the uterus, along the breast of the child, until one foot was secured, which was brought down in contact with the presenting arm. By proper traction and external manipulation, with this alone, the delivery could have been effected; but, supposing the difficulty might be lessened, the other foot was sought and brought down. By gentle traction upon the lower extremities, the arm readily returned, version was easily effected, and the woman promptly delivered of a dead child. At the time the feet were brought down, and before traction and manipulation were attempted, the patient complaining considerably, partial anæsthesia was produced by chloroform, which not only controlled the suffering, but, to some extent, the tonic contraction or rigidity of the parts.

Considerable irritability of the system was manifested after the delivery—the pulse 130 to the minute, with restlessness and pain. Brandy and morphine were administered; and, as no hemorrhage or other serious difficulty presented, the patient was left with the nurse for the evening. She has been as well as could be expected since, and gives hope of speedy recovery.

While eminent obstetricians, among them Tyler Smith, recommend eviceration, and the delivery without turning, he considered the latter less painful and injurious to the mother, and would prefer it, even when the death of the child is certain.

Dr. Boring mentioned a case in which the woman had been in labor three days, with arm presentation. When called, he found her very much exhausted; so much so, that, after consultation, turning was considered impracticable. The dimensions of the child were reduced by eviceration, and it withdrawn from the womb. It was supposed the child had been dead forty-eight hours.

After the delivery, the woman did well for thirty-six hours, when symptoms of puerperal peritonitis were manifested. She sank rapidly under the violence of the inflammation, and died in a few days. He expressed himself decidedly in favor of turning, when the arm presents, let the circumstances otherwise be what they may.

Dr. Armstrong said he was struck with the remarks of Dr. Boring, and with the similarity of termination to a case he had met with, on account of another difficulty. In company with another physician, he had been called to a woman in labor forty-eight hours. The water had escaped, and pains ceased twelve hours previously. She was much exhausted—quick frequent pulse. Upon examination, the os was found fully dilated, and no resistance or hindrance to the delivery, connected with the uterus itself, discovered; but, on proceeding further with the examination, it was ascertained that a disproportion in the size of the head and pelvis manifestly existed; so much so, indeed, that a passage was impossible. Without delay, the cranium was punctured, and a large quantity of fluid, followed by the natural contents, was discharged. The child was then brought away by gentle traction, and no further trouble experienced in the management of the case for twenty-four hours. At the end of this time, fever, with other symptoms of peritonæo-hysteritis, came on, and progressed rapidly to a fatal termination.

Dr. Word had seen three cases of congenital hydrocephalus, but, in every instance, they resulted favorably to the mother by evacuating the accumulated fluid. He agreed fully with Dr. Boring in the opinion that, in arm presentation, turning is preferable to eviceration. He also expressed pleasure in being able to corroborate the statement of Dr. O'Keefe, that chloroform has a relaxing influence upon the soft parts in labor. He had noticed this result occasionally in his obstetrical practice.

